# Childhood scurvy: an unusual cause of refusal to walk in a child

**DOI:** 10.1186/s12969-015-0020-1

**Published:** 2015-06-11

**Authors:** J. T. Alqanatish, F. Alqahtani, W. M. Alsewairi, S. Al-kenaizan

**Affiliations:** Department of Pediatrics, King Abdulaziz Medical City and King Saud Bin Abdulaziz University for Health Sciences, National Guard Health Affairs, Riyadh, Saudi Arabia; Department of Dermatology, King Abdulaziz Medical City and King Saud Bin Abdulaziz University for Health Sciences, National Guard Health Affairs, Riyadh, Saudi Arabia

**Keywords:** Scurvy, Pseudovasculitis, Arthritis, Vitamin C (ascorbic acid)

## Abstract

Scurvy, or vitamin C deficiency, is rarely presented to a rheumatology clinic. It can mimic several rheumatologic disorders. Although uncommon, it may present as pseudovasculitis or chronic arthritis. Scurvy still exists today within certain populations, particularly in patients with neurodevelopmental disabilities, psychiatric illness or unusual dietary habits.

Scurvy presentation to the rheumatologist varies from aches and mild pains to excruciating bone pain or arthritis. Musculoskeletal and mucocutaneous features of scurvy are often what prompts referrals to pediatric rheumatology clinics. Unless health care providers inquire about nutritional habits and keep in mind the risk of nutritional deficiency, it will be easy to miss the diagnosis of scurvy. Rarity of occurrence as compared to other nutritional deficiencies, combined with a lack of understanding about modern-day risk factors for nutritional deficiency, frequently leads to delayed recognition of vitamin C deficiency.

We report a case of scurvy in a mentally handicapped Saudi child, who presented with new onset inability to walk with diffuse swelling and pain in the left leg. Skin examination revealed extensive ecchymoses, hyperkeratosis and follicular purpura with corkscrew hairs, in addition to gingival swelling with bleeding. Clinical diagnosis of scurvy was rendered and confirmed by low serum vitamin C level. The patient did extremely well with proper nutritional support and vitamin C supplementation.

It has been noticed lately that there is increased awareness about scurvy in rheumatology literature. A high index of suspicion, together with taking a thorough history and physical examination, is required for diagnosis of scurvy in patient who presents with musculoskeletal symptoms. Nutritional deficiency should also be considered by the rheumatologist formulating differential diagnosis for musculoskeletal or mucocutaneous complaints in children, particularly those at risk.

## Background

In the 21st century, the most devastating nutritional problem for many is not lack of nutrients, but rather excessive calorie intake leading to obesity and other metabolic syndromes. However, despite modernization among almost all societies, nutritional deficiencies still occur in certain populations. Children who suffer neurodevelopmental disabilities, psychiatric illness or those with unusual dietary habits are prone to vitamin C deficiency.

Scurvy has been known since ancient times but the relation between scurvy and its prevention by citrus fruits was described for the first time by Sir James Lind on 1753. Subsequently, ascorbic acid was first isolated in 1928 [1].

Vitamin C is an essential exogenous vitamin because the l-gulonolactone oxidase gene is non-functional so it prevents coding for the enzyme required to convert glucose into ascorbic acid in humans [2]. Ascorbic acid is involved in the hydroxylation of collagen, the biosynthesis of carnitine and norepinephrine, the metabolism of tyrosine, and the amidation of peptide hormones [3]. Hence the clinical presentations of vitamin C deficiency are directly connected to its various uses by the body.

Vitamin C has a half-life of 10 to 20 days. However, signs of deficiency generally develop after 1 to 3 months of inadequate vitamin C intake. Clinical features of vitamin C deficiency may overlap with systemic diseases such as rheumatologic, infectious, or hematologic disorders. Musculoskeletal and mucocutaneous manifestations in children with scurvy are numerous, characterized by perifollicular petechiae, bruising and ecchymoses, gingival inflammation and bleeding, alopecia, and bone pain, skeletal muscle degeneration, and arthritis [4].

Given the present-day rarity of this disease, the consideration of scurvy as a diagnosis is often overlooked by physicians, leading to extensive laboratory and radiographic testing and unnecessary delays in diagnosis and treatment [5].

We report a case of scurvy in a mentally handicapped Saudi child who presented with new onset inability to walk with diffuse swelling and pain in the left leg. The patient’s parents gave their written consent for publication of this report and use of the photos in the manuscript.

## Case presentation

A 12 year-old boy with global neurodevelopmental delay was referred to the rheumatology clinic with worsening musculoskeletal pain and refusal to walk for two months. He also had skin eruption, restlessness and severe anemia for five months. The skin eruption started as a purpuric rash over his lower extremities that never faded away. A few courses of prednisone resulted in no improvement. Shortly after the onset of rash, he developed coombs’-positive hemolytic anemia that required three transfusions of packed red blood cells (PRBCs). Two months prior to his presentation to the clinic, he began refusing to walk, for which he was seen by a pediatrician and orthopedic surgeon. Radiographs showed no fracture, so physiotherapy was advised. Physiotherapy was interrupted by worsening pain and swelling over the left leg. He was seen by a neurologist after the onset of his symptoms and was given clonazepam to treat restlessness.

There was no preceding history of trauma, fever, change in level of consciousness, or seizures. The patient also presented with no constitutional symptoms, no bleeding from body orifices, and no change in bowel habits. Preceding upper respiratory tract infection (URTI) and gastroenterology or genitourinary symptoms were also absent.

The patient was borne of a twin pregnancy that was ended at 28 weeks’ gestation. Apgar at 1 and 5 min after birth was 5 and 6 respectively. He stayed ventilated in NICU for 2 months, and developed retinopathy of prematurity and retinal detachment, subsequently losing vision in the left eye. He was known to have global developmental delay with non-revealing neurological or metabolic work up. The delay had therefore been attributed to complications of prematurity. Although the family had a normal diversified diet, the child was exclusively milk-fed. He was not able to eat or chew hard food because of bad dental hygiene. Family history was unremarkable for neurological, metabolic or bleeding problems.

### Initial examination

The patient came to the clinic on a wheelchair and was pale. Tachycardiac HR was 120 beats/minute, afebrile, with blood pressure of 113/70 mmHg. He was agitated but conscious, alert and oriented. His weight was 21.5 kg (<5th percentile), height was 134.5 cm (25th percentile), and BMI was11.88 kg/m^2^ (underweight).

**Fig. 1:**
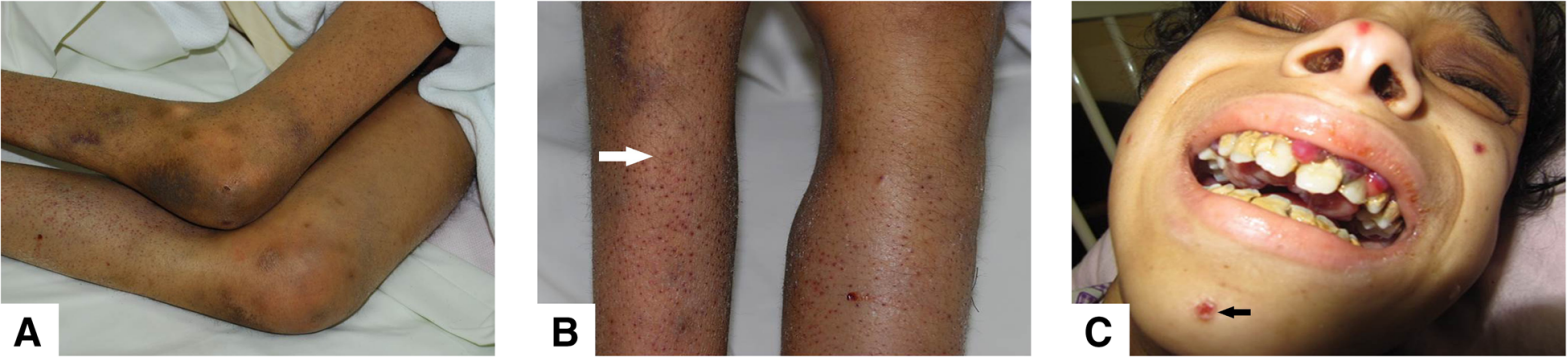
Patient at presentation. **a** Knee flexion contractures and ecchymoses, **b** hyperkeratosis and follicular purpura with corkscrew hairs (*white arrow*), **c** hemorrhagic gingivitis and neurotic excoriations (*black arrow*)

There was hard swelling over the left thigh, which was mildly tender and diffusely swollen, and the patient refused to ambulate. He maintained 90°flexion contracture over both knees (Fig. 1a). It was difficult to assess his motor system because of his pain. Skin examination revealed extensive ecchymoses, follicular hyperkeratosis and follicular purpura with corkscrew hairs (white arrow), and neurotic excoriations (black arrow) (Fig. 1a, b and c). Gingival swelling and intermittent fragility bleeding were present (Fig. 1c). Red reflex was absent over the left eye, normal over right. There were no abnormal findings on chest, cardiovascular and abdominal examination.

Immediate admission for evaluation was arranged and he had been evaluated by specialists in areas including hematology, dermatology, neurology, gastroenterology and nutrition. Work up revealed very low hemoglobin, 56 g/L, so urgent PRBCs transfusion was given. Platelet count was 371 × 10^9^ /L and reticulocyte count was 4 %. Peripheral smear showed moderate anisopoikilocytosis. His erythrocyte sedimentation rate was 44 mm/hour (reference range: 0–15 mm/hour) at the initial presentation. Hemoccult blood was negative. Bleeding disorder work up including prothrombin time, partial thromboplastin time, international normalized ratio (INR), platelet aggregation tests, and factor assay for factors VII, XIII and IX were normal. C-reactive protein, coagulation profile, and chemistry including calcium, phosphorus, and magnesium were also normal. Serum iron level was 2.16 μmol/L (reference range 11.6-31.3), vitamin C level was 5.7 μmol/L (reference range: 28.4-85.2), 25-OH vitamin D was 92.2 nmol/L(reference range: 75–250), red blood cell (RBC) folate was 281 nmol/L (reference range: 362–1616), Vitamin B12 was 468 pmol/L (reference range: 138–652). Radiographs revealed a questionable right fibular fracture that was not confirmed on a technetium 99 m bone scan. Ultrasound revealed a large intramuscular hematoma of different ages over the left thigh, measuring 5 × 20 cm in anteroposterior and craniocaudal diameter respectively.

Ascorbic acid (vitamin C) supplement was initiated at 500 mg twice daily for 3 days, then 300 mg daily for 2 weeks and maintained on100mg daily for 3 months. By 4 weeks, the patient was ambulating freely, and skin lesions and gingival swelling improved. Three months later, he came to the clinic walking.

Hemoglobin was 133 g/L, and albumin 43 g/L. A repeat vitamin C level was 75 μmol/L (reference range: 28.4-85.2).

## Discussion

Humans lack the ability to synthesize vitamin C, because of mutation in the gene coding for l-gulonolactone oxidase, the enzyme required for the biosynthesis of vitamin C [6]. However, vitamin C is an essential micronutrient. Scurvy occurs as a result of decreased vitamin C consumption or absorption. The prevalence of scurvy in children is not known, but certain populations are at risk. The two largest cohorts of scurvy cases in children have been reported by Noble et al., who presented 23 case studies of scurvy in children with restricted diets, including children with autism, developmental delay, and cerebral palsy [7], and Ratanachu-Ek et al., who reviewed 28 cases of scurvy in Thai children, almost all of whom presented with inability to walk. Of the cases reviewed by RatanachuEk et al., 89 % were supplemented with ultra heat treated (UHT) milk [8].

The earliest manifestations of scurvy are nonspecific constitutional symptoms, such as asthenia, anorexia, and weight loss. Musculoskeletal manifestations may be the presenting symptoms in scurvy; in 80 % of cases, the manifestations of scurvy include arthralgia, myalgia, hemarthrosis, and muscular hematomas [9]. As in our case, inability to walk is a very common presenting musculoskeletal symptom in children with scurvy [8].

Poorly formed collagen leads to dystrophic or corkscrew hairs, gingival hyperplasia, and weakened blood vessel walls, causing bleeding in the skin, joints, and other organs [10]. Follicular hyperkeratosis and perifollicular hemorrhages are also common, particularly over the lower limbs, as was a prominent feature in our case. Subperiosteal hematomas may be palpable as painful swellings over the distal end of the femur and tibia. Intramuscular hematomas may cause compartment syndrome [9]. Our patient had large, uncomplicated intramuscular hematoma that resolved spontaneously.

Laboratory test abnormalities in scurvy are nonspecific; anemia is frequent manifestation. Our patient had severe hemolytic anemia that required multiple blood transfusions. In severe cases, anemia can lead to cardiac hypertrophy and high output heart failure [6].

The causes for anemia in scurvy could be multifactorial, resulting from blood loss, concomitant vitamin deficiencies, and decreased iron absorption [11]. An elevated erythrocyte sedimentation rate and C-reactive protein level can be seen in some cases [12]. In our case, the sedimentation rate was mildly elevated, which could cause confusion with an inflammatory process.

Radiographic changes may include osteonecrosis, osteopenia, and cortical thinning with periosteal proliferation. Many signs have been reported in the literature, such as the Frankel sign (zone of calcification at the margin of growth plate), Wimberger sign (calcification around the epiphysis) and scurvy line (lucency adjacent to metaphyseal sclerotic line) [13]. In our case, all of these findings were absent. We speculate that a good level of vitamin D halted these changes.

A meticulous history, including dietary history and physical examination, can help to reach the diagnosis of scurvy. Usually, work up is not necessary to confirm diagnosis of scurvy.

Vitamin or mineral deficiencies other than vitamin C deficiency may accompany scurvy, therefore the prudent clinician will also check for concomitant micronutrient deficiencies, testing levels of zinc, iron, folate, and vitamin B12. In our case, RBC folate and serum iron were low.

Known risk factors for scurvy in children are oral aversions, cerebral palsy, developmental delay, and autism. Our patient had developmental delay as a result of complicated prematurity.

Scurvy is preventable disease. The daily intake of vitamin C must be no lower than 10 mg/day and the body pool no smaller than 350 mg to avoid scurvy [2]. The recommended daily intake is variable according to the child’s age [14]. Treatment of scurvy is based on replenishing the level of vitamin C to counteract the symptoms. There is no established regimen for vitamin C supplementation in scurvy. Supplementation with 1 g/day of oral vitamin C for 2 weeks is the usual treatment [9]. However, other regimens have been tried, such as 100 to 200 mg/day for a longer period [4]. Once vitamin C deficiency has been treated, prevention and correction of underlying conditions are required in order to prevent recurrence.

Following treatment with vitamin C, spontaneous bleeding, oral symptoms and constitutional symptoms begin to improve within days, while bony changes and ecchymoses may take several weeks to resolve [12]. Overall improvement of symptoms after the administration of ascorbic acid is a confirmatory approach for the diagnosis.

## Conclusion

Scurvy can present to rheumatologists with different manifestations, which mandates careful assessment of the diet for each child attending rheumatology clinic. The possibility of scurvy in modern times should not be overlooked, in particular among susceptible populations. Coexistence of other nutrient deficiency is not unusual and, when appropriate, screening for such deficiencies should be considered. Most of the time, musculoskeletal complaints in scurvy are accompanied by cutaneous manifestations that may guide the diagnosis. Confirmation of scurvy is made by low serum ascorbic acid level. However, the most cost effective indicator of disease is simply resolution of the manifestations following replenishing the level with ascorbic acid supplements. Awareness of vitamin C deficiency in children who present with musculoskeletal pain to rheumatology clinic is necessary to avoid invasive and unnecessary investigations and to preserve medical resources.
